# Milk Proteins, Peptides, and Oligosaccharides: Effects against the 21st Century Disorders

**DOI:** 10.1155/2015/146840

**Published:** 2015-02-19

**Authors:** Chia-Chien Hsieh, Blanca Hernández-Ledesma, Samuel Fernández-Tomé, Valerie Weinborn, Daniela Barile, Juliana María Leite Nobrega de Moura Bell

**Affiliations:** ^1^Department of Human Development and Family Studies (Nutritional Science & Education), National Taiwan Normal University, No.162, Section 1, Heping East Road, Taipei 106, Taiwan; ^2^Instituto de Investigación en Ciencias de la Alimentación (CIAL, CSIC-UAM, CEI UAM+CSIC), Nicolás Cabrera 9, 28049 Madrid, Spain; ^3^Food Science Department, University of California, Davis, One Shields Avenue, Davis, CA 95616, USA; ^4^Foods for Health Institute, University of California, Davis, One Shields Avenue, Davis, CA 95616, USA

## Abstract

Milk is the most complete food for mammals, as it supplies all the energy and nutrients needed for the proper growth and development of the neonate. Milk is a source of many bioactive components, which not only help meeting the nutritional requirements of the consumers, but also play a relevant role in preventing various disorders. Milk-derived proteins and peptides have the potential to act as coadjuvants in conventional therapies, addressing cardiovascular diseases, metabolic disorders, intestinal health, and chemopreventive properties. In addition to being a source of proteins and peptides, milk contains complex oligosaccharides that possess important functions related to the newborn's development and health. Some of the health benefits attributed to milk oligosaccharides include prebiotic probifidogenic effects, antiadherence of pathogenic bacteria, and immunomodulation. This review focuses on recent findings demonstrating the biological activities of milk peptides, proteins, and oligosaccharides towards the prevention of diseases of the 21st century. Processing challenges hindering large-scale production and commercialization of those bioactive compounds have been also addressed.

## 1. Introduction: Role of Milk in Human Health

Milk, as the first food for mammals, supplies all the energy and nutrients needed for the proper growth and development of the neonate. For all mammalians, the consumption of milk ends at the weaning period with the exception of humans that continue consuming milk throughout their life. Milk and derived dairy products are considered an important constituent of a balanced diet. Moreover, it is a source of many bioactive components, such as high-quality proteins, lipids, carbohydrates, lactose, vitamins, minerals, enzymes, hormones, immunoglobulins, and growth factors, among others. These components not only help meeting human nutritional requirements, but also play a relevant role in preventing various disorders such as hypertension and cardiovascular diseases [1], obesity [2], osteoporosis [3], dental caries [4], poor gastrointestinal health [5], colorectal cancer [6], ageing [7], and others [8].

Milk proteins supply nitrogen and amino acids to young mammals and possess multiple physiological properties in the intact form. Moreover, studies carried out in the past decades have demonstrated the role of these proteins as a source of biologically active peptides. Bioactive peptides are inactive within the sequence of the parent protein but, once released by* in vitro* processing conditions or by* in vivo* gastrointestinal digestion, are capable of acting as regulatory compounds exerting a positive impact on body functions and ultimately promoting health benefits to the consumer [9].

Human milk is undoubtedly the most complete source of nourishment for the newborn. Breastfed infants have been shown to be less susceptible to diseases (i.e., diarrhea and respiratory diseases) than those that were not breastfed. This protective effect, which was previously attributed to human milk antibodies, is today strongly correlated with the presence of complex oligosaccharides (OS), the third most abundant component of human milk [10]. Human milk is composed of OS in concentrations varying according to different stages of lactation: 20–23 g/L in colostrum and 12–14 g/L in mature milk [11], being even more abundant than proteins (12 g/L) [12]. Human milk oligosaccharides (HMO) are complex sugars having 3 to 20 monosaccharide units [13] that are not digestible by human enzymes [14]. These compounds have important functions related to the newborn's development and health at local and systemic levels, including prebiotic probifidogenic effects and antiadherence of pathogenic bacteria [15], brain development [16], and immunomodulatory properties [17], among others.

In the last fifty years, chronic disorders have become the leading cause of morbidity and mortality in industrialized countries, with increasing incidence also observed in developing countries. Chronic disorders include cardiovascular and neurological diseases, stroke, cancers, immune disorder and chronic respiratory disease, obesity, diabetes, and metabolic syndrome [18]. In Europe, 87% of all deaths occur due to chronic diseases and the number of people affected is expected to rise considerably over the next few decades. The majority of chronic diseases are caused by risk factors which are mostly preventable. Diet and lifestyle are two environmental factors that strongly affect these diseases; thus modifications of these habits are becoming a new strategy for disease prevention/treatment.

The aim of this paper is to review the recent literature on the physiological effects of proteins, peptides, and oligosaccharides with special emphasis on animal and human trials. Other aspects such as the limited availability of* in vivo *studies demonstrating the biological activities of OS from bovine and caprine milk and the current challenges associated with the recovery and commercial production of these compounds have also been addressed.

## 2. Impact of Milk Proteins and Peptides on the 21st Century Diseases

### 2.1. Milk-Derived Peptides against Cardiovascular Diseases

Cardiovascular diseases (CVD) have become the leading cause of morbidity and mortality worldwide, representing an important medical and public health issue [19]. Although earlier studies associated the consumption of whole milk with higher incidence of CVD, it has been demonstrated that milk contains a plethora of bioactive substances which may contribute to the prevention of most of the risk factors of CVD [20]. Recently, bioactive milk peptides have gained interest because of their notable antihypertensive, antioxidant, anti-inflammatory, and hypocholesterolaemic effects. In this section, the most current scientific information regarding* in vitro* and* in vivo* studies on the role of milk proteins-derived peptides on CVD is summarized and discussed.

#### 2.1.1. Milk Peptides with Antihypertensive Activity

Epidemiological studies suggest that the dietary intake of milk and dairy foods is related to decreased risk of hypertension [21]. In addition to their high mineral content (e.g., calcium, potassium, and magnesium) that can lower blood pressure [22], other milk components, such as proteins and their hydrolyzed products, have been also linked to the antihypertensive effect of milk and dairy products. Angiotensin-converting enzyme (ACE) is a multifunctional enzyme that acts as one of the main regulators of blood pressure. Thus, ACE inhibition is currently considered as one of the best strategies for hypertension treatment. Most biologically active peptides generated from milk proteins have demonstrated ACE inhibitory activity. In the last two decades, antihypertensive effects of some of these peptides have been evaluated in spontaneously hypertensive rats (SHR) and hypertensive humans, and the peptide sequences, doses, and maximum decreases of systolic blood pressure (SBP) have been summarized in several reviews [23–25]. The hydrolyzate obtained by the action of pepsin on casein, containing the *α*_s1_-casein-derived peptides RYLGY and AYFYPEL, has been patented and commercialized under the name of Lowpept by its antihypertensive properties demonstrated in both SHR [26] and hypertensive humans [27] (Table 1). Pepsin has been also used to hydrolyze whey protein lactoferrin, with the release of peptides containing ACE activity and ACE-dependent vasoconstriction inhibitory properties [28]. Antihypertensive effects in SHR after short-term and long-term treatments have been also observed for those peptides [29, 30]. Trypsin is another gastrointestinal enzyme used to release the antihypertensive peptide *α*_s1_-casein peptide f(23–34) from casein during the manufacture of the commercial ingredient peptide C12 [31, 32] (Table 1). In addition to the use of gastric and pancreatic enzymes, alone or in combination, to produce antihypertensive peptides, the use of food-grade enzymes derived from microorganisms has become common for the release of peptides with demonstrated SBP lowering effects in SHR [33–36].

Milk fermentation is another strategy to produce antihypertensive peptides by the proteolytic action of lactic acid bacteria on milk proteins. The most representative peptides are those derived from *β*-casein and identified in sour milk fermented by* Lactobacillus helveticus *and* Saccharomyces cerevisiae* (Calpis, Table 1). These tripeptides, with sequences VPP and IPP, have demonstrated an ability to exert potent decreasing effects on the SBP of SHR [37, 38]. A number of clinical trials have been conducted to confirm their antihypertensive properties in humans although controversial results have been found. Three meta-analyses performed with the published data of 17 [39], 12 [40], and 28 [41] clinical trials have reported an average decrease in SBP of 5.1, 4.8 mm, and 1.7 mm of Hg, respectively. However, no effects were found in Dutch and Danish subjects consuming fermented milk containing peptides VPP and IPP [42, 43]. A recent meta-analysis including 18 trials has reported higher antihypertensive effects for these two tripeptides in Asian than in Caucasian people [44]. Those findings suggest that genetics and/or dietary patterns might exert an important influence on the antihypertensive effects of peptides IPP and VPP. Similarly, the age has been described as another major influencing factor [45]. With the evidence presented to date, the European Food Safety Authority (EFSA) Panel on Dietetic Products, Nutrition and Allergies (NDA) [46] concluded that there are no sufficient data to establish a cause/effect relationship between the consumption of peptides VPP and IPP and the control of hypertension, and further studies are thus required. Other peptides derived from *β*-casein during milk fermentation with* Enterococcus faecalis*, in which sequences are LHLPLP and HLPLP, have also shown antihypertensive effects in SHR [47]. In recent studies, fermented milk with* Lactococcus lactis* NRRLB-50571 and NRRLB-50572 has presented important SBP, diastolic blood pressure (DBP), and heart rate-lowering effects in SHR [48, 49] although the peptides responsible for the activity have not been identified.

Accumulating evidence built in animal and clinical studies is currently available on the antihypertensive activity of milk-derived peptides. However, much work is still needed. Identification of the active form reaching the target organs and elucidation of its bioavailability after oral ingestion and its complete mechanism of action are two of the main aspects required to be deeply investigated in the future to support health claims.

#### 2.1.2. Antioxidant and Anti-Inflammatory Milk-Derived Peptides

Oxidative stress is one of the main responsible factors for the initiation or evolution of CVD. The search of natural antioxidants providing additional benefits to the endogenous antioxidant defense system is gaining interest [50]. Among food-derived peptides with antioxidant properties without harm side effects, those derived from milk proteins are most frequently studied. The majority of the studies carried out to characterize antioxidant peptides derived from casein and whey proteins have only used* in vitro *chemical assays [51, 52]. However, their limited similarity to physiological conditions makes the* in vitro *assays very restrictive, and reported effects need to be confirmed by animal models and/or human trials. Nevertheless, to date, just few* in vivo* trials have been carried out to demonstrate the antioxidant effects of milk-derived peptides related to benefits on cardiovascular health. Zommara et al. [53] reported the antiperoxidative action of fermented milk on rats fed a vitamin-E deficient diet. The consumption of fermented milk by healthy subjects has been also demonstrated to lower the levels of oxidized low-density lipoprotein, isoprostanes, and the glutathione redox ratio. Improvements of total plasma antioxidant activity and of the resistance of the lipoprotein fraction to oxidation have resulted in enhanced antiatherogenicity [54]. The compounds responsible for the observed effects have not been identified yet, although milk peptides liberated during fermentation process might have a crucial role. Thus, further studies focused on evaluating the potential of milk-derived peptides as antioxidant at cardiovascular level should be of great relevance.

Chronic inflammation is another responsible factor for the development of CVD. The downregulation of cytokines involved in the inflammation-associated endothelial dysfunction by food components, including peptides, may delay or alleviate inflammation, thus exerting favorable effects against CVD [55]. A recent study using lipopolysaccharide- (LPS-) stimulated mouse macrophages has reported the ability of a yak casein hydrolyzate to reduce the secretion of proinflammatory cytokines and the production of nitric oxide and to scavenge free radicals, suggesting a potential role as preventive agent against inflammation related disorders [56]. To date, only one human trial has been conducted to demonstrate the anti-inflammatory properties of milk peptides. This study reported an improvement in the vascular function through modulation of the glucose levels and inflammation and oxidative stress biomarkers after the consumption of the commercial whey derived peptide NOP-47 by healthy individuals [57]. This finding opens a new door towards searching of new milk-derived peptides with antioxidant and anti-inflammatory activity.

#### 2.1.3. Hypocholesterolaemic Milk Peptides

Blood lipids are represented in various forms including total cholesterol, triglycerides, lipoproteins (high-density lipoproteins or HDL, low-density lipoproteins or LDL, and very-low-density lipoproteins or VLDL), and free fatty acids. An inappropriate ratio of these lipids is one of the most important risk factors for developing CVD. Therefore, CVD therapy/prevention strategies focus on reaching an optimal lipid balance in order to achieve a positive cardiovascular health. Those therapies aim at increasing the physiological levels of desirable lipids (e.g., HDL cholesterol) while reducing the others associated with atherogenic functions (e.g., LDL cholesterol, triglycerides). Milk proteins, mainly whey proteins and derived hydrolyzates or peptides, have been reported to exert hypocholesterolaemic effects in different animal models. The ingestion of whey protein was correlated with a significant reduction of total cholesterol levels in rats fed with cholesterol-free and cholesterol-enriched diets [58, 59]. Nagaoka et al. [60] have reported similar effects for a *β*-lactoglobulin tryptic hydrolyzate administered to rats fed with a diet rich in cholesterol. The hydrolyzate reduced total cholesterol and increased HDL cholesterol and fecal steroid excretion. The fragment f(71–75) of this whey protein, known as lactostatin, with sequence IIAEK, has been reported as the main factor responsible for the observed effects [60]. *β*-Lactotensin, another *β*-lactoglobulin peptide, released by chymotrypsin hydrolysis, decreased total cholesterol, LDL, and VLDL cholesterol content in mice fed with a cholesterol-enriched diet [61]. Although the mechanism of action of those peptides has not been completely elucidated, preliminary results suggest a key role played by the amino acid composition [50]. Further studies are clearly needed to corroborate those results. The exact mode of this hypocholesterolaemic action needs to be determined in clinical trials.

### 2.2. Milk-Derived Hydrolyzates and Peptides on Intestinal Health

The gastrointestinal tract (GIT) serves as a specialized interface between the body and the external environment. The GIT is strategically covered by a monolayer of specially designed epithelial cells continually exposed to a high concentration of food components and substances along the gut luminal surface. Hence, the modulator effect of the diet on GIT functions has been accepted as essential for maintaining and improving the general health of the host [62]. Interestingly, more than 70% of the current “food for specified health uses products” (FOSHU) are related to GIT functions [63].

Dairy proteins, hydrolyzates, and peptides have been demonstrated to transform the dynamics of mucus mainly via influencing the mucin secretion and expression and the number of goblet cells. In* ex vivo *preparations of rat jejunum, casein hydrolyzates increased mucin secretion [64, 65]. The *β*-casein derived peptide *β*-casomorphin 7 produced the same effects which have been suggested to be mediated by interaction with opioid receptors. Also, this peptide has been reported to stimulate the expression of mucin* Muc2* and* Muc3* genes in rat intestinal DHE cells and* MUC5AC* gene in human intestinal HT29-MTX cells [66]. Another *β*-casein fragment, f(94–123), identified in commercial yoghurt, also had the ability to increase the mucin output and the mRNA levels of* MUC2* and* MUC4* genes in HT29-MTX cells [67]. Casein and whey proteins hydrolyzates have been reported to be a source of peptides with capacity to induce mucin secretion and* MUC5AC* gene expression in HT29-MTX cells [68]. Among these peptides, the *α*_s1_-casein fragments f(143–149) and f(144–149) and the *β*-lactoglobulin fragment f(102–105) known as *β*-lactorphin were suggested as the major peptides responsible for the observed effects.

A few* in vivo *studies have also pointed out the regulation of the protective mucus layer by dairy proteins and products thereof. Rats fed with a diet based on casein hydrolyzates, as the exclusive source of nitrogen, were found to enhance their endogenous nitrogen flow and expression of mucin genes* Muc3* and* Muc4* in the small intestine and colon, respectively [69]. Plaisancié et al. [67] reported the capacity of the *β*-casein fragment f(94–123), once orally ingested by rats, to upregulate the* Muc2, Muc4, rat defensing 5* and* lysozyme* mRNA transcripts expression, the goblet cells recounts, and the number of crypts containing Paneth cells in the rat small intestine. In the dextran sulphate sodium- (DSS-) induced model of rat colitis, the studies of Sprong et al. [70] and Faure et al. [71] demonstrated the gut-protective effects exerted by a cheese whey protein diet and a diet supplemented with Thr, Ser, Cys, and Pro residues, respectively. Moreover, this protection has been reported for a whey protein isolate and *α*-lactalbumin hydrolyzate against chemical-induced ulcerative gastric lesions [72, 73].

Enhancement of the mucosal immune response is also a dietary modulating strategy of the defense systems protecting the GIT. Animal models have proved the improvement of the mucosal immunity by promotion of gut-related immunoglobulin (Ig) levels after ingestion of lactoferrin or its derived peptides, lactoferricin and lactoferrampin [74, 75]. Likewise, immunomodulatory effects have been reported for a trypsin casein hydrolyzate in newborn calves [76] and casein phosphopeptides (CPPs) and peptides released from* Lactobacillus helveticus *R389-fermented milk in mice [77, 78]. Furthermore, Kitamura and Otani [79] demonstrated that ingestion by healthy humans of CPPs-enriched cakes induced an increase in the faecal IgA content, suggesting a positive effect on mucosal immunity.

Oxidative and inflammatory imbalances are both involved in the etiology of several human chronic gut-related disorders such as ulcerative colitis and Crohn's disease. The search of natural preventive treatments against these imbalances is being prompted [80, 81]. Whey protein has been suggested to exert beneficial effects through enhancement of antioxidant enzymes and downregulation of both oxidative markers and proinflammatory cytokines [82]. These protective findings were found in animal [83, 84] and humans trials [85, 86]. The whey-derived peptide caseinomacropeptide has been proven to have protective properties in the 2,4,6-trinitrobenzene sulphonic acid (TNBS) and DSS-induced model of rat ileitis and colitis, through immunomodulation of the regulatory T helper cells activation and interleukin secretions [87, 88]. Turbay et al. [89] demonstrated, in the TNBS-induced murine colitis model, the anti-inflammatory effects exerted by *β*-casein hydrolyzates generated by the cell envelope-associated proteinase of* Lactobacillus delbrueckii *ssp.* lactis *CRL 581. However, peptides released and responsible for the observed bioactivity have not been identified yet.

### 2.3. Milk Proteins and Peptides against Metabolic Disorders

Diabetes mellitus is considered one of the most common metabolic disorders and one of the major health problems worldwide. It affects almost 6% of the world's population, with type 2 diabetes representing approximately 90–95% of the diagnosed cases [90]. Diet and lifestyle interventions are the preferred treatment strategies for this metabolic disorder, with pharmacotherapy being prescribed only if supervised lifestyle intervention fails [91]. Epidemiological evidence supports that consumption of milk and dairy foods is associated with a lower incidence of type 2 diabetes. These beneficial effects on metabolic and inflammation factors linked to diabetes and insulin resistance have been also demonstrated by cell and animal models, being multiple milk components, such as calcium, medium-chain fatty acids, linoleic conjugated acid, lactose, citrate, proteins, and peptides characterized as the main responsible factors for the observed effects acting through different mechanisms of action [92].

During the ingestion of a meal, the presence of nutrients at gastrointestinal level stimulates the secretion of two incretins hormones, the glucagon-like peptide-1 (GLP-1) and the glucose-dependent insulinotropic polypeptide (GIP). Both hormones are implicated in the stimulation of the insulin secretion from the pancreatic *β*-cells, secretion of gastric and pancreatic enzymes, and modulation of gut motility and nutrient absorption, allowing the clearance of the absorbed glucose [93]. Type 2 diabetes is characterized by different disorders including progressive dysfunction of pancreatic cells, insulin resistance, and augmented production of hepatic glucose [94]. Continuous intravenous administration of GLP-1 has been demonstrated to normalize blood glucose levels in diabetic subjects [95]. However, the rapid degradation of this hormone by the enzyme dipeptidyl peptidase-IV (DPP-IV) and its consequent inactivation makes this type 2 diabetes treatment strategy impracticable. Currently, specific DPP-IV inhibitors are thus incorporated to GLP-1 analogues in new oral therapies against this metabolic disease [96].

Diet supplementation with whey protein is currently under preclinical and clinical trials as a promising alternative in the prevention and/or treatment of type 2 diabetes and related diseases [97, 98]. Several mechanisms of action have been suggested for whey protein, including the stimulation of insulin release, improvement of glucose tolerance in diabetic patients, reduction of body weight, and modulation of gut hormones such as cholecystokinin, leptin, and GLP-1 [99]. In the last years, the role of peptides released during the transit of whey proteins through the GIT on the observed effects has been hypothesized [100]. Cell culture and animal models have been used to confirm this hypothesis. A dose-dependent insulinotropic activity of whey protein hydrolyzates has been observed in a cell-based coculture using pancreatic BRIN-BD11 cells and Caco-2 cells monolayers [101]. These authors also observed that the oral administration of the hydrolyzates to obese mice evoked an improvement of blood glucose clearance, reduction of hyperinsulinemia, and restoration of the pancreatic capacity to secrete insulin in response to glucose. The main mechanism of action suggested for these hydrolyzates is the DPP-IV inhibitory activity exerted by the peptides contained in them [102]. Among the bioactive peptides described to date, sequences derived from *β*-lactoglobulin IPA and IPAVF are the most potent as DPP-IV inhibitors [103, 104]. Another *β*-lactoglobulin fragment with sequence VAGTWY has been also demonstrated to exert hypoglycemic effects in the oral glucose tolerance test in mice [105]. Likewise, both* in vitro *DPP-IV inhibitory and* in vivo *hypoglycemic effects have been reported for peptides released from caseins [106]. Recent* in silico *studies have shown that both caseins and whey proteins might serve as precursors of DPP-IV inhibitory peptides because of the high number of fragments contained within them that match DPP-IV inhibitory sequences [107, 108]. Thus, this research area holds a great potential, and currently a number of investigations are focused on the identification of new milk proteins-derived peptide with capacity to prevent diabetes and associated metabolic syndromes.

### 2.4. Chemopreventive Role of Milk Proteins and Peptides

Cancer is the second leading cause of mortality worldwide, and its incidence will continue rising in the next few years in spite of the important advances achieved in the development of cancer therapies. It has been estimated that, by 2020, approximately 15 million new cancer cases will be diagnosed, and 12 million cancer patients will die [109]. It is well known that 35% of cancer deaths are attributed to diet and its food components [110]. However, cell culture and animal and human trials results have shown that an important number of food constituents can lower cancer risk and even sensitize tumor cells against anticancer therapies [111]. In the last few years, food proteins and derived peptides have become one of the food components with the most promising preventive properties against cancer initiation, promotion, and progression stages [112].

Among the milk proteins, lactoferrin and its derived peptide lactoferricin are the most studied. For both compounds, their antioxidant, immunomodulatory, and anti-inflammatory activities are closely linked to their protective effects against cancer (Table 2). Lactoferrin acts by inducing apoptosis, inhibiting angiogenesis, and modulating carcinogen metabolizing enzymes, in addition to its antioxidant and immunomodulatory properties [113]. Moreover, lactoferricin has shown potent anticancer properties in different cell lines, including breast, colon, fibrosarcoma, leukemia, and oral and ovarian cancer cells, without harming normal lymphocytes, fibroblasts, or endothelial or epithelial cells [114]. Also, animal models have confirmed the beneficial properties of this milk-derived peptide. The possible mechanism of bovine lactoferricin in anticarcinogenesis has been shown to be related to its ability to induce apoptosis. It is its strongly cationic nature that allows this peptide to target negatively charged cancer cells with the outer membrane [115]. The suppressed ability in angiogenesis of bovine lactoferricin was* in vitro* and* in vivo* demonstrated to contribute to its chemopreventive properties [116]. A significant inhibition of tumor growth and of liver and lung metastasis was reported after subcutaneous administration of bovine lactoferricin in both spontaneous and experimental metastasis mice models [117]. Similar results were observed after subcutaneous treatment and repeated injections of this peptide on Meth A fibrosarcoma xenografts mice and established neuroblastoma xenografts, respectively [118, 119].

*α*-Lactalbumin is a whey protein with anticancer properties which has been reported when it forms a complex with oleic acid known as “human alpha-lactalbumin made lethal to tumor cells, HAMLET” or “bovine alpha-lactalbumin made lethal to tumor cells, BAMLET.” It has been recognized that both protein and fatty acid are required to show cytotoxic activity against cancer cells [115]. Treatment of cancer cells with HAMLET provokes morphological changes typical of apoptotic cells through caspase activation and causes mitochondrial permeability transition resulting in mitochondrial swelling, loss of mitochondrial membrane potential, and cytochrome c release [120]. These authors also found that this complex induced autophagic cell death and changes in the proteasome structure and function. Similar effects resulting from chromatin condensation and cell shrinkage have been observed after treatment of cancer cells with the complex BAMLET. The efficacy of both complexes has been shown to be influenced by the type of cancer cell line [120]. In the last years, the therapeutic effects against bladder cancer have been studied in animal models as preliminary step for BAMLET use in human trials. It has been demonstrated that intravesical administration of HAMLET delays tumor progression in a murine bladder cancer model although no preventive effects on tumor formation were observed [121].

Intact caseins have not been characterized as chemopreventive proteins but they have been suggested as an important source of peptides with anticancer properties. CPPs are able to bind calcium, to inhibit cell proliferation, and to induce apoptosis of intestinal tumor HT-29 and AZ-97 cells through activation of voltage-activated calcium channels, which mediate the calcium flood according to the depolarization state of the cell [122]. However, in differentiated epithelial intestinal cells, a protective effect from programmed cell death is observed after treatment with these peptides [123]. *β*-Casomorphin 7 and *β*-casomorphin 5, two casein-derived sequences with opioid properties, have shown antiproliferative and cell cycle arresting activities on breast and colon cancer cells [115, 124, 125]. It has been suggested that these effects are mediated by interaction with specific opioid and somatostatin receptors although further studies confirming this mode of action are needed.

## 3. Impact of Milk Oligosaccharides on Human Health

Despite the important role of HMO in infant health, the limited supply of human milk has hindered its use in commercial infant formula [126] and in large-scale clinical trials. Presumably, the health benefits provided by HMO to infants could be extended to humans of all ages if alternative sources of these complex OS are identified [127]. In that view, the need of finding other sources of human-like OS has prompted the identification, characterization, and quantification of unknown OS present in many other types of milk and their respective industrial streams [128, 129].

### 3.1. Alternative Sources of Oligosaccharides: Major Sources of Nonhuman Milk Oligosaccharides and Their Industrial Effluents

Increasing interest on plant- and lactose-derived OS has been observed in the past decade as an alternative source for complex HMO. Some of these OS include galacto-OS (GOS), fructo-OS (FOS), and lactulose, among others [130]. These indigestible OS are considered prebiotics due to their ability to confer health benefits to the host through the selective growth and activity of commensal bacteria [131]. One such example is inulin, an oligofructan with D-fructofuranosyl *β*(1-2) links that cannot be broken down by human digestive enzymes, thus exerting several intestinal physiological effects that contribute to the host health. GOS, commonly produced by transgalactosylation of lactose by *β*-galactosidases, are another example of a current available source of OS for use by the infant formula industry.

Despite the fact that some health promoting effects, such as improved bifidogenic activity, have been attributed to some of those OS [131], little similarity has been observed between commercially available GOS and HMO, except that they are both built on a lactose core [127]. GOS and FOS are composed of a simple linear core, being devoid of structures having high biological activity such as fucose, sialic acid, and* N*-acetyl glucosamine. Because GOS and FOS do not possess the intrinsic structural complexity observed in HMO, it is expected that domestic farm animals and their processing streams, such as whey permeate from cheese manufacturing, can be a source of OS more similar to the ones present in human milk [132].

World milk production is almost entirely derived from cattle (83%), buffaloes (13%), goats (2%), sheep (1%), and camels (0.3%) (http://www.fao.org/agriculture/dairy-gateway/milk-production/dairy-animals/en/#.VA95gvldXXs). Considering that cow milk accounts for 83% of the world milk production, the enormous interest of the scientific community to identify, quantify, and characterize the OS present in cattle milk and their industrial byproducts is not surprising. A comprehensive review by Urashima et al. [132] shows that approximately 25 bovine milk OS (BMO) structures had been characterized before 2011. The development of advanced analytic techniques, such as several mass spectrometric methods and hydrophilic interaction liquid chromatography-high performance liquid chromatography, has enabled significant improvement in the identification of new BMO; as many as 40 BMO have been characterized [133, 134].

The low concentration of BMO makes it challenging to identify and characterize these compounds when compared with HMO. The OS concentration can reach values as high as 0.7–1.0 g/L in bovine colostrum or can be detected as just trace amounts in bovine milk [135], being much lower than the OS concentration in human milk. Caprine milk is another type of milk, which contains complex OS similar to HMO. The discovery of the presence of fucosylated and sialylated OS that are considered as prebiotics and which have the ability to reduce pathogen adherence to the intestine wall has opened up translational opportunities to human health [136]. Approximately 37 caprine milk OS (COS) have been identified, of which nearly half of them have had their structural complexity elucidated. Similar to bovine milk, COS are present in very small concentrations when compared with HMO. However, they have been found in concentrations of 0.25–0.3 g/L, which is 4-5 times higher than BMO [137].

From those two alternative sources of HMO-like OS (BMO and COS), industrial streams arising from cheese manufacturing and production of whey protein concentrates (WPC) and isolates (WPI) have been considered as a more realistic source of OS for future commercialization [129, 138]. Considering the enormous worldwide production of whey (180–190 × 10^6^  tonnes/year; http://www.adpi.org/Portals/0/PDF/09Conference/TAGEAFFERTSHOLT.pdf) and the fact that the major industrial application of whey to produce WPC and WPI generates a new byproduct containing the target OS, the development of economically feasible processes to recover these compounds represents a key step in enabling the large-scale production of OS.

### 3.2. Biological Activities of Oligosaccharides

While a wide range of biological functions has been attributed to HMO, less information is available regarding the biological activities of BMO and COS. The limited availability of large quantities of OS with high degree of purity can be inferred by the limited number of* in vivo* studies with those compounds, with the majority of milk OS biological activities being described by* in vitro* studies. Recent reports of some of the biological activities of HMO, BMO, and COS are reported in Table 3.

#### 3.2.1. Prebiotic Activity

One of the main features of HMO is that they can only be consumed by very specific bacteria strains that possess the appropriate set of enzymes to cleave their complex structure. This prebiotic effect is associated with improved health outcomes. A prebiotic is “a selectively fermented ingredient that allows specific changes, both in the composition and/or in the activity in the gastrointestinal microflora, conferring benefits upon host well-being and health” [139]. Because HMO are only partially digested in the small intestine, they can reach the colon intact where they selectively stimulate the development of bifidogenic flora. A recent study has demonstrated the bifidogenic effect of major fucosylated and sialylated HMO when fed as a sole source of carbon to 25 major isolates of the human intestinal microbiota [140]. Most of the* Bifidobacteria* spp. and* Bacteroides* spp. were able to consume those OS and to produce short chain fatty acids, while common pathogenic bacteria were not able to grow on those OS.* In vitro* biological activities of HMO have been supported by* in vivo* studies. One of the newest publications in this topic demonstrated the ability of 2-fucosyllactose and 3-fucosyllactose to selectively increase some intestinal bacteria populations like* Barnesiella*, the major bacterial genus in mice [141], being this effect correlated with reduced level of colitis.

Prebiotic activities of COS, recovered from caprine whey, have been evaluated by* in vitro* studies [142]. The purified COS fraction favored the development of* Bifidobacterium *spp. and produced short chain fatty acids such as lactic and propionic acids but presented no inhibition of* Staphylococcus aureus* and* Escherichia coli* grown in human faeces.

#### 3.2.2. Antipathogenic Activity

A second feature of OS is the ability to reduce pathogen biding to the intestinal mucosa. The intestinal mucosa is heavily glycosylated and covered with complex glycans including glycoproteins, glycolipids, and mucins, among others [143, 144]. Bacteria and viruses are able to recognize certain types of fucosylated and sialylated OS and adhere to them [130], therefore acting as anti-infective agents. Milk OS are also fucosylated and sialylated so bacteria and viruses, in presence of OS, will attach less to intestinal cells. The ability of pathogens to bind to specific OS seems to be intrinsically correlated with their structure. Neutral OS containing HexNAc block adhesion of pathogens that cause diarrhea (*Vibrio cholerae*) and pneumonia (*Streptococcus pneumoniae*) [15, 145], while neutral fucosylated OS have been shown to inhibit adhesion of other pathogens (i.e.,* Campylobacter jejuni* and diarrheagenic* E. coli*) that cause gastrointestinal disorders [146]. Acidic OS containing sialic acid are able to block adhesion of* Helicobacter pylori*, which causes peptic ulcers and other gastric diseases [147],* Staphylococcus aureus*, and* Clostridium botulinum *[148].

Recent* in vitro* studies have demonstrated that BMO also possess antibacterial properties as observed for HMO. BMO from colostrum permeate proved to be effective in protecting HEp-2 cells from enteropathogenic* E. coli*,* Cronobacter sakazakii*, and* Salmonella enterica *serovar* typhimurium* [149]. It has also been demonstrated that BMO can inhibit the pili-mediated adhesion of* Neisseria meningitidis in vitro* [150]. Several studies have demonstrated the inhibition of the attachment of enteric pathogens such as* E. coli* and* Campylobacter jejuni* and noroviruses with HMO [151]. This effect has also been demonstrated by* in vivo* studies in which isolated HMO were fed to suckling mice before and after infection with enteropathogenic* E. coli*. Mice that received HMO significantly reduced colonization of this species compared with untreated controls [152].

#### 3.2.3. Anti-Inflammatory Activity

OS have been also considered as anti-inflammatory agents due to their prebiotic activities and their ability to act as receptors of microorganisms.* In vivo* studies have demonstrated that COS possess anti-inflammatory properties towards the development of experimental colitis in rats. Pretreatment of the rats with isolated COS reduced the typical signs of induced colitis, including less anorexia, better body weight gain, and less macroscopic intestinal lesions, among others [153]. Similar results were observed by Lara-Villoslada et al. [154], where COS were shown to play an important role in intestinal protection and repair after a damage caused by DSS in rats.

## 4. Future Prospects

Milk has long been recognized as a source of macro- and micronutrients. Recent identification of many important biologically active substances on milk and its derivatives has attracted much attention from the scientific community. Not only are many of these bioactive compounds associated with growth, but they also confer many health benefits that might support disease prevention. Milk proteins and peptides are usually well tolerated and demonstrate oral bioavailability. In this view, they have the potential to act as health promoting ingredients and to participate in auxiliary therapies to boost overall success in chronic diseases. However, this research area is only at its beginning and more peptides with physiological effects are to be discovered in the future. Confirming the health benefits of these bioactive compounds requires the design of clinical trials based on metabolomic genomics, proteomics, transcriptomics, and epigenetic data, in order to explore new biomarkers related to the observed health benefits.

While larger data for* in vivo* biological activities of milk and peptides is observed, the same is not observed for OS. To date, few studies have demonstrated the safety and efficacy of OS supplementation [155, 156]. The reduced number of biological activities evaluated for BMO and COS reveals the challenges associated with the production of OS in adequate quantities and purity needed for clinical trials. The development of new synthetic pathways to produce highly purified OS and of large-scale processes to recover those OS from their respective industrial streams will likely improve the elucidation of their biological activities and determine their safety and efficacy in clinical trials with humans. Moreover, the development of more environmentally friendly processes that are also economically feasible not only will enable the production of a new generation of prebiotics but will address environmental issues associated with the disposal of OS-containing waste streams and poor economic viability of our food industry.

## Figures and Tables

**Table 1:** Commercial milk products containing peptides with proven antihypertensive activity.

Commercial name	Obtention process	Protein source	Active sequence(s)	Publication number [reference]
Peptide C12	Hydrolysis with trypsin	*α*_s1_-Casein	FFVAPFPEVFGK	JP62270533 [31]
Biozate	Hydrolysis with trypsin	Whey proteins	Whey peptides	US6998259 [157]
Lowpept	Hydrolysis with pepsin	*α*_s1_-Casein	RYLGY, AYFYPEL	WO2005012355 [158]
Calpis	Fermentation	*β*-Casein	VPP, IPP	US5449661A [37, 38]
Evolus	Fermentation	*β*-Casein	VPP, IPP	US6972282 [159]

**Table 2:** Chemopreventive properties of lactoferrin and its derived peptide lactoferricin against cancer demonstrated by cell culture experiments and animals models.

Type of cancer	Animal species/protein-peptide	Cell line/animal model	Effects/mechanisms of action	Reference

Breast cancer	Human lactoferrin	MDA-MB-231 cells	Inhibition of cell growth Cell cycle arrest	[160]
	Bovine lactoferrin	4T1 xenograft Balb/c mice	Improvement of tamoxifen chemopreventive effects Downregulation of proinflammatory cytokines	[161]
	Bovine lactoferrin-oleic acid complex	MCF-7 cells	Inhibition of proliferation Induction of apoptosis	[162]
	Bovine lactoferricin	MCF-7, T-47D, and MDA-MB-435 cells	Cytotoxic activity Induction of apoptosis	[163]

Colon cancer	Camel lactoferrin	HCT-116 cells	Inhibition of cell proliferation Antioxidant activity Inhibition of DNA damage	[164]
	Bovine lactoferrin	Caco-2 xenograft mouse model	Inhibition of tumor growth	[165]
	Bovine lactoferrin-oleic acid complex	HT-29 cells	Inhibition of proliferation Induction of apoptosis	[166]
		C26 cells	Cytotoxic activity	[118]
		Caco-2 cells	Inhibition of cell proliferation	[166]
			Cell cycle arrest by downregulation of cyclin E1	
	Bovine lactoferricin	Ultraviolet-irradiated Caco-2 cells	Reduction of DNA damage Cell cycle arrest by downregulation of cyclin E1	[167]
		Colo-35 and HT-29 cells	Cytotoxic activity/induction of apoptosis	[163]

Cervical cancer	Bovine lactoferrin	HeLa cells	Inhibition of cell growth Induction of nuclear accumulation of Smad-2	[168]

Fibrosarcoma	Bovine lactoferricin	Meth A cells	Cytotoxic activity Tumor cell membrane disruption	[118]

Head and neck cancer	Human lactoferrin	Squamous carcinoma O12 tumor bearing mice	Reduction of tumor Immunomodulatory effects	[169]

Hepatocarcinoma	Bovine lactoferrin-oleic acid complex	HepG2 cells	Inhibition of proliferation Induction of apoptosis	[162]

Leukemia	Bovine lactoferricin	THP-1 human monocytic leukemic cells	Induction of apoptosis Activation of ROS generation and Ca^2+^/Mg^2+^-dependent endonucleases	[170]
		Jurkat T leukemia cells	Induction of apoptosis by triggering mitochondrial swelling and release of cytochrome c Induction of cell membrane permeabilization	[163, 171]
			Activation of ROS generation and caspase-3 and caspase-9 activity Reduction of DNA methyltransferases expression	[172]

Lymphoma		Raji and Ramos Burkitt's B-lymphoma cells	Induction of apoptosis Stimulation of DNA fragmentation, chromatin condensation, and nuclear disintegration	[114]
		Ramos B-lymphoma cells xenografts in SCID/beige mice	Extension of survival of mice	[114]
	Bovine lactoferricin	A20 cell lymphomas in syngeneic Balb/c mice	Tumor necrosis and regression of the tumors Induction of long-term specific cellular immunity	[173]
		B16-BL6 melanoma and L5178Y-ML25 lymphoma cells metastasis models in syngeneic mice	Inhibition of tumor metastasis in lung	[117]

Lung cancer	Bovine lactoferrin	A549 cells Transgenic mice overexpressing hVEGF-A165	Downregulation of proinflammatory cytokines Suppression of tumor development	[174]

Melanoma	Bovine lactoferricin	B16F10 cells	Cytotoxic activity	[118]
		Spontaneous B16-BL6 metastasis models in syngeneic mice	Inhibition of tumor metastasis in lung	[117]

Nasopharyngeal carcinoma	Human lactoferrin	5–8F, CNE2, and HONE1 cells Xenograft Balb/c mice	Suppression of tumorigenesis through inhibition of the AKT pathway	[175]

Neuroblastoma	Bovine lactoferricin	Human MYCN-amplified and non-MYCN-amplified neuroblastoma cells	Cytotoxic activity Destabilization of the cytoplasmic membrane Activation of caspase-6, caspase-7, and caspase-9	[119]
		SH-SY-5Y neuroblastoma xenografts in nude rats	Reduction of the tumor growth	[119]

Oral cancer	Bovine lactoferricin	Oral squamous carcinoma SAS cell	Induction of apoptosis Cleavage of caspase-3 and poly-ADP ribose polymerase Phosphorylation of extracellular signal-regulated kinase and c-Jun N-terminal kinase/stress activated protein kinase	[176]

Ovarian cancer	Bovine lactoferricin	Skov3 and Caov3	Cytotoxic activity Induction of apoptosis	[163]

**Table 3:** Biological activities of human, bovine, and goat oligosaccharides.

Microorganisms/animals	Molecule used	Dose	Duration/details	Outcome measured	Reference
*Bifidobacterium* spp., *Bacteroides* spp., *Clostridium *spp., *Lactobacillus* spp., *Enterococcus *spp., *Streptococcus* spp., *Staphylococcus* spp., *Enterobacter* spp., and *Escherichia coli *	HMO (2′-FL, 3′-FL, LDFT, 3′-SL and 6′-SL)	0.5–2 g/L	48 hrs OS incubation	SCFA quantification, bacterial growth, and OS consumption	[140]
Mice	HMO (2′-FL and 3′-FL)	500 mM, starting with 5 mL, increasing by 2.5 mL every 3 d reaching a daily amount of 25 mL on day 20	From day 1 to day 20 after birth	Bacterial amount, colitis signs	[141]
Bacteria from human feces	Pooled GOS		During incubation	Bacterial amount	[142]
Mice	Pooled HMO	15 mg/day	One day before and after infection with EPEC	Intestinal colonization of EPEC	[152]
HEp-2 cells	Pooled BMO from colostrum	20 mg/L of total carbohydrate in culture	During incubation	Adherence inhibition	[149]
Bovine thyroglobulin and human salivary agglutinin glycoproteins	Pooled HMO and BMO	40 g/L	During incubation	*Neisseria meningitidis* Pili attachment	[150]
Rats	Pooled GOS	500 mg/(kg∗d)	2 days before and 6 days after induced colitis	Colonic damage	[153]

HMO: human milk oligosaccharides; FL: fucosyllactose; LDFT: lacto-difucosyl-tetraose; SL: sialyllactose; GOS: galacto-oligosaccharides; BMO: bovine milk oligosaccharides.
